# Anticancer Properties of the Antipsychotic Drug Chlorpromazine and Its Synergism With Temozolomide in Restraining Human Glioblastoma Proliferation *In Vitro*

**DOI:** 10.3389/fonc.2021.635472

**Published:** 2021-02-26

**Authors:** Silvia Matteoni, Paola Matarrese, Barbara Ascione, Mariachiara Buccarelli, Lucia Ricci-Vitiani, Roberto Pallini, Veronica Villani, Andrea Pace, Marco G. Paggi, Claudia Abbruzzese

**Affiliations:** ^1^ Cellular Networks and Molecular Therapeutic Targets, Proteomics Unit, IRCCS - Regina Elena National Cancer Institute, Rome, Italy; ^2^ Center for Gender Specific Medicine, Oncology Unit, Istituto Superiore di Sanità, Rome, Italy; ^3^ Department of Oncology and Molecular Medicine, Istituto Superiore di Sanità, Rome, Italy; ^4^ Fondazione Policlinico Universitario A. Gemelli IRCCS, Institute of Neurosurgery, Catholic University School of Medicine, Rome, Italy; ^5^ Neuro-Oncology, IRCCS-Regina Elena National Cancer Institute, Rome, Italy

**Keywords:** glioblastoma, antipsychotic drugs (APDs), drug repurposing and repositioning, cancer stem cells (CSC), neurospheres, drug synergism, clinical trials

## Abstract

The extremely poor prognosis of patients affected by glioblastoma (GBM, grade IV glioma) prompts the search for new and more effective therapies. In this regard, drug repurposing or repositioning can represent a safe, swift, and inexpensive way to bring novel pharmacological approaches from bench to bedside. Chlorpromazine, a medication used since six decades for the therapy of psychiatric disorders, shows *in vitro* several features that make it eligible for repositioning in cancer therapy. Using six GBM cell lines, three of which growing as patient-derived neurospheres and displaying stem-like properties, we found that chlorpromazine was able to inhibit viability in an apoptosis-independent way, induce hyperdiploidy, reduce cloning efficiency as well as neurosphere formation and downregulate the expression of stemness genes in all these cell lines. Notably, chlorpromazine synergized with temozolomide, the first-line therapeutic in GBM patients, in hindering GBM cell viability, and both drugs strongly cooperated in reducing cloning efficiency and inducing cell death *in vitro* for all the GBM cell lines assayed. These results prompted us to start a Phase II clinical trial on GBM patients (EudraCT # 2019-001988-75; ClinicalTrials.gov Identifier: NCT04224441) by adding chlorpromazine to temozolomide in the adjuvant phase of the standard first-line therapeutic protocol.

## Introduction

Glioblastoma (GBM, glioblastoma multiforme) is the most frequent and severe adult malignant brain tumor. The best available therapeutic approach toward newly diagnosed GBM patients, *i.e.* surgical ablation followed by radiotherapy plus concomitant and adjuvant chemotherapy with temozolomide (TMZ), is associated with a median survival of 15 months (1). GBM’s highly aggressive, chemo-resistant and relapse-prone behavior is mainly attributed to its intra-tumor molecular heterogeneity associated with unpredictable genetic drift under therapeutic pressure (2). Such an adverse scenario prompts for the identification of novel therapeutic approaches even by using repurposed/repositioned drugs that, when supported by robust evidence, represent an attracting alternative to novel drugs, being safer, less expensive, and characterized by a shorter timeframe from laboratory to the clinics.

We focused our attention on chlorpromazine (CPZ, Largactil, Thorazine), the first member of the tricyclic drugs phenothiazines, a medication used since six decades in the treatment of psychiatric disorders. This molecule acts as an antagonist of the brain dopamine receptor D2 (DRD2), thus decreasing post-synaptic dopamine stimulating activity (3, 4). DRD2 is highly expressed in GBM, mainly in glioma-initiating cells, where it regulates homeostasis, enhancing resistance to hypoxia and increasing cellular plasticity (5). Furthermore, several reports show that CPZ can inhibit cancer cell growth through several mechanisms (6–15). In addition, epidemiological data suggest a reduction of cancer risk in psychiatric patients treated with CPZ or related antipsychotic compounds (16, 17), and anecdotal reports of favorable GBM evolution in psychiatric patients treated with neuroleptic medications have been published (16, 18).

We evaluated the ability of CPZ to affect several GBM cellular parameters *in vitro*, using a large number of human GBM cell lines, *i.e.* the anchorage-dependent cell lines T98G, U-87 MG, and U-251 MG as well as three patient-derived, anchorage-independent neurospheres characterized for their ability to display a glioma stem-like cell behavior (19). In addition, hTERT-immortalized retinal pigment epithelial cells (RPE-1) (20), a non-cancer cell line from neuro-ectodermal origin, were also used in selected assays.

Here we investigate, for the first time to our knowledge, the synergistic effect between CPZ and TMZ, the reference drug for first-line GBM clinical treatment, in inhibiting GBM cell growth in either anchorage-dependent or -independent, patient-derived stem-like neurospheres.

## Materials and Methods

### Cell Lines

Anchorage-dependent cell lines T98G, U-251 MG and U-87 MG were cultured as previously reported (21). Anchorage-independent TS#1, TS#83 and TS#163 neurospheres are patient-derived cell lines from surgical samples classified according to WHO 2016 (22), isolated and cultured in order to enrich them with glioma stem cells, as described (19, 23, 24). Human hTERT-immortalized retinal pigment epithelial cells (RPE-1) (20) were a kind gift from Giulia Guarguaglini, CNR, Rome, Italy.

T98G, U-251 MG and U-87 MG are from the laboratory of one of the authors (L.R.V.). Their authentication was performed by short tandem repeat (STR) profiling, which resulted in ≥80% match for eight loci as per interrogation of the ATCC STR profiling database. TS#1, TS#83, and TS#163 neurospheres have been defined as glioma stem-like cells according to established criteria (25, 26). TS#83 grow partially in an anchorage-dependent fashion.

All cell lines were Mycoplasma-free and used for a maximum of 20 passages.

### Drugs

CPZ was purchased, as “Largactil”, from Teofarma S.R.L., Valle Salimbene (PV), Italy, as a 25 mg/ml solution (78 mM). TMZ was purchased from Selleckchem (Houston TX, USA) and diluted in DMSO as a 150 mM solution.

### Cell Viability Assay

This assay was performed as previously described (27). Briefly, 5 × 10^3^ cells were seeded in a 96-well plate and treated with CPZ for 48 h; then the relative number of viable cells was determined by CellTiter-Glo Luminescent Cell Viability Assay (Promega, Madison, WI), analyzed by means of a GLOMAX 96 Microplate Luminometer (Promega) and dose–response curves were generated (Prism v5, GraphPad Software Inc., San Diego, CA). When synergy between TMZ and CPZ was assayed, cells were initially treated with TMZ for 96 h and then CPZ was added for further 48 h at a fixed dose, approximately corresponding to inhibitory concentration IC10. Control samples were treated with the same final concentration of the respective drug solvent(s) (DMSO for TMZ and PBS for CPZ). A dose–response curve was also calculated using TMZ as a single agent for each GBM cell line; in these experiments, cells were exposed to the drug for 6 d.

### Fluorescence Microscopy

**Figure 1:**
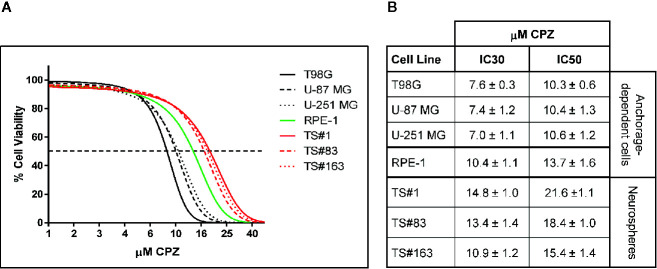
CPZ reduces cell viability in GBM cells. **(A)**. Representative dose–response curves of all cell lines treated with CPZ for 48 h are shown. GBM anchorage-dependent cell lines are represented by black lines, while RPE-1 by a green line and neurospheres by red ones. **(B)**. Table showing μM CPZ concentrations corresponding to the IC30 and IC50 calculated for each cell line. Values are expressed as mean ± SE.

For analysis of nuclear morphology by fluorescence microscopy, treated cell lines were exposed to CPZ at the concentrations reported as IC30 in **Figure 1B** for 48 h and control cells to an equal volume of solvent (PBS). Cells were then fixed in 4% paraformaldehyde, stained with Hoechst 33258 (Sigma-Aldrich, St. Louis, MO; 1 mg/ml in PBS) and mounted in glycerol/PBS (ratio 1:1, pH 7.4). Images were acquired by intensified video-microscopy (IVM) with an Olympus fluorescence microscope (Olympus Corporation, Tokyo, Japan), equipped with a Zeiss charge-coupled device camera (Carl Zeiss, Oberkochen, Germany). Control cells were exposed to an equal volume of solvent (PBS).

### Colony-Forming Assay

This assay was performed as described (27). Briefly, anchorage-dependent GBM cells were plated at a concentration of 2–3 × 10^2^ cells/well in 6-well plates and treated with increasing doses of CPZ for 48 h. When synergy between TMZ and CPZ was assayed, cells were initially treated with a sub-optimal dose of TMZ for 96 h, then CPZ was added for further 48 h at a sub-optimal dose. Cells were then washed, cultured for additional 12 d and subsequently stained using a 5% crystal violet solution to assess the colony number. Control samples were treated with the same final concentration of the respective drug solvents, PBS for CPZ and DMSO for TMZ.

### Neurosphere Formation Assay

This assay was performed as described (27). Briefly, TS#1, TS#83 and TS#163 cells were plated at 2.5 × 10^5^ cells/well in a 6-well plate and treated with increasing doses of CPZ for 48 h. Alternatively, to evaluate the synergy between CPZ and TMZ, cells were treated with the respective drug solvents, PBS for CPZ and DMSO for TMZ, or a fixed dose of TMZ for 96 h and then CPZ was added for further 48 h. Cells were then mechanically dissociated into single cell suspension, counted, diluted at the appropriate concentration and re-seeded in triplicate into new 6-well plates (5 × 10^2^ cells/well) in the absence of drugs. After 20 further days, neurosphere-forming efficiency was examined by inverted microscopy.

### RNA Extraction and RT-PCR

Both anchorage-dependent cells and neurospheres were treated with a dose of CPZ corresponding to their respective IC30 for 24 h. Control samples were treated with the same final concentration of PBS. Treated and control cells were harvested, and total RNA was extracted using miRNeasy Extraction Kit (QIAGEN, Hilden, Germany). RNA concentration was determined using the NanoDrop 1000 spectrophotometer, then was reverse-transcribed into cDNA with High Capacity cDNA Reverse Transcription Kit (ThermoFisher Scientific, Waltham, MA). To quantify gene expression, qRT-PCR was performed using SYBR Green in a QuantStudio 6 Flex Real-Time PCR System (ThermoFisher Scientific) and CT values were normalized to GAPDH. All RT-PCR data were analyzed using the 2^−ΔΔCT^ method. Values represent the fold changes referred to the respective value for control cells, arbitrarily reported as 1.0.

### Flow cytometry—Apoptosis

For apoptosis evaluation, treated cell lines were exposed to TMZ (96 h) and CPZ (48 h), or their combination, at the lowest concentrations considered synergistic based on the viability analysis. Control cells were exposed to an equal volume of solvent(s) (PBS and/or DMSO). Apoptosis was quantified using a fluorescein isothiocyanate (FITC)-conjugated Annexin V (AV) and propidium iodide (PI) detection kit (Marine Biological Laboratory, Woods Hole, MA, USA). This assay enables identifying both early (AV positive/PI negative) and late apoptotic or necrotic (PI positive) cells. Alternatively, cell death was evaluated by incubating cells with 1 μm Calcein-AM (ThermoFisher Scientific) at 37°C for 30 min. In live cells, the non-fluorescent Calcein-AM is converted to a green-fluorescent dye, whereas dead cells, with compromised cell membranes, do not retain Calcein, thus not displaying green fluorescence.

### Flow Cytometry–Cell Cycle

For cell cycle analysis, treated cell lines were exposed to CPZ at the concentrations reported as IC30 in **Figure 1B** for 48 h and control cells to an equal volume of solvent (PBS). Cells were then fixed in cold 70% ethanol for 30 min at 4°C. After washing in PBS, cells were incubated with ribonuclease (50 µl of a 100 µg/ml stock of RNase), to ensure that only DNA, not RNA, was stained, and PI (200 µl from a 50 µg/ml stock solution).

We measured the forward scatter (FS) and side scatter (SS) to identify single cells. Pulse processing (pulse area vs. pulse width) was used to exclude cell doublets from the analysis.

Samples were analyzed by collecting FL2 red fluorescence in a linear scale at 620 nm. Acquisition was performed on a dual-laser FACSCalibur flow cytometer (BD Biosciences, San Jose, CA) and at least 30,000 events/sample were run in low mode and acquired. Data were analyzed using the Cell Quest Pro and ModFit software (BD Biosciences).

### Statistical Analysis

Unless otherwise specified, all tests were done in triplicate. Results are expressed as mean ± standard error. Differences between two groups were analyzed using the Student’s two-tailed t test. Asterisks denote statistical significance (*p < 0.05; **p < 0.01; ***p < 0.001). When statistical analyses were performed among more than two groups, data were analyzed by One-way ANOVA test followed by Tukey’s Multiple Comparisons Test (Prism v5).

We used the algorithm described by Fransson et al. (28), to assess if the effect of the combination of CPZ and TMZ was synergistic, additive or antagonist. The effect of either compounds used as single agents compared with that of the drug combination, is expressed as Combination Index (CI). A CI value <0.8 indicates synergism; a CI value between 0.8 and 1.2 indicates an additive effect, while a CI value >1.2 indicates antagonism.

## Results

### CPZ Reduces GBM Cell Viability

**Figure 1A**, depicts the effect on viability of a 48 h-exposure to increasing doses of CPZ in six different GBM cell lines cells. The graph refers to the anchorage-dependent cells T98G, U-87 MG, and U-251 MG (solid, dashed, and dotted black lines, respectively) and the TS#1, TS#83, and TS#163 neurospheres (solid, dashed, and dotted red lines, respectively). In addition, the non-cancer RPE-1 cells (20) were also assayed for their susceptibility to CPZ (solid green line).

CPZ markedly affected cell viability in all six GBM cell lines. Drug doses required to achieve IC30 and IC50 (the amount of substance able to inhibit *in vitro* a given biological process by 30 or 50%, respectively) were lower for anchorage-dependent GBM cells and appeared higher in stem-like neurospheres. Interestingly, RPE-1 cells, also growing in an anchorage-dependent fashion, appeared less sensitive to CPZ than anchorage-dependent GBM cells, but more sensitive than neurospheres, which are characterized by slow replication rates and the need of an enriched stem cell culture medium. IC30 and IC50 for all these cell lines are reported in **Figure 1B**.

### CPZ Induces Cell Cycle Alterations and causes Hyperdiploidy in GBM Cells

**Figure 2:**
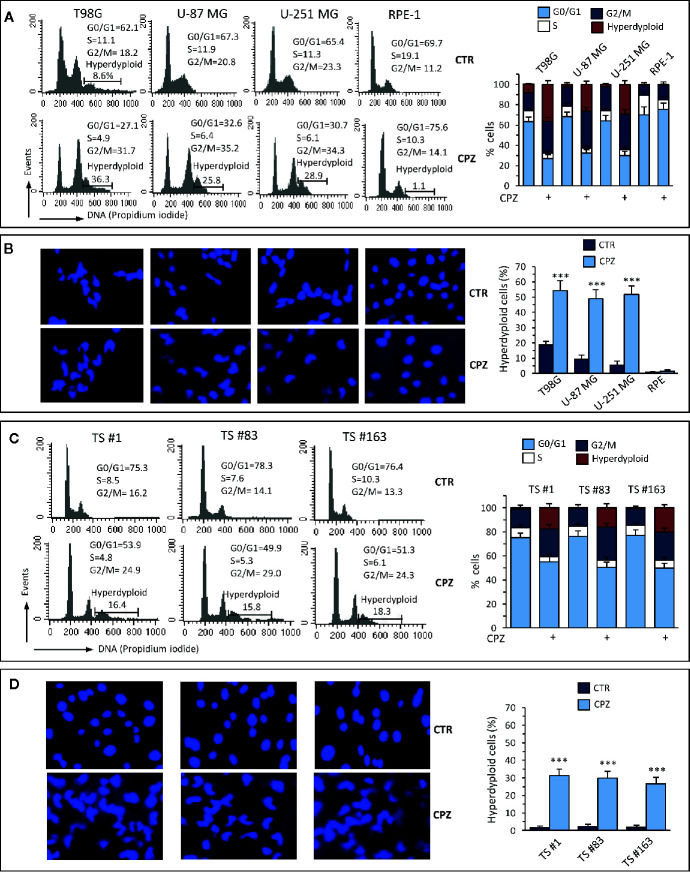
CPZ induces alterations of the cell cycle and hyperdiploidy in GBM cells. Anchorage-dependent GBM cell lines [panels **(A)** and **(B)**] or neurospheres [panels **(C)** and **(D)**] underwent treatment with CPZ at the calculated IC30 for 48 h. Flow cytometry analysis of the cell cycle. Left panels **(A)**, **(C)**. Histograms obtained in a representative experiment. Numbers represent the percentage of cells in the different phases of the cell cycle or hyperdiploid cells. Right panels **(A)**, **(C)**. Bar graphs showing mean ± SE of data obtained from three independent experiments. Left panels **(B)**, **(D)**. Representative micrographs of the analysis of nuclear morphology performed after cell staining with Hoechst 33258. Right panels **(B)**, **(D)**. Bar graphs showing the quantification of hyperdiploid cells performed by counting at least 50 cells from 10 different fields observed with a 40× objective. Data are reported as mean ± SE of data obtained from three independent experiments. Statistical significance is referred toward the Control (*** p < 0.001).

Several reports claim for CPZ the ability of protecting from apoptosis in either normal neural cells exposed to toxic stimuli (29, 30) or malignant glioma cells (10). According to these studies, apoptotic cell death should not be involved in the drug-induced decrease in GBM cell viability and/or proliferation rate. To investigate this topic, we treated GBM cells with CPZ for 48 h before analyzing cell cycle parameters *via* propidium iodide (PI) FACS analysis. As shown in **Figure 2A**, all the anchorage-dependent GBM cells treated with CPZ exhibited a significant increase in the percentage of cells in G2/M phase of the cell cycle, as it has been previously suggested for the sole U-87 MG cell line (10). Furthermore, no increase of the hypodiploid peak, characteristic of apoptosis, was detectable in any of these cell lines after CPZ treatment, where, by contrast, an increased number of hyperdiploid cells were apparent. Of note, RPE-1 cells undergoing the same treatment did not display any hyperdiploid phenotype.

Analysis of nuclear morphology in CPZ-exposed GBM cells confirmed the presence of abnormal nuclei. Also in this case, RPE-1 cells did not show any apparent hyperploidy or alteration of nuclear morphology (**Figure 2B**). The experimental results obtained employing the three neurospheres show that cell cycle modifications and nuclear aberrations after exposure to CPZ were qualitatively comparable with those obtained with anchorage-dependent GBM cells, although at a lesser extent (**Figure 2C, D**).

These data confirm the ability of CPZ to interfere with the G2/M phase of the cell cycle and induce aberrant mitotic segregation in GBM cells, both phenomena able to induce cell death *via* an apoptosis-independent mechanism. The anchorage-dependent T98G cell line displayed a portion of hyperdiploid cells at the baseline, as described (31). Nonetheless, treatment with CPZ significantly increased the number of T98G hyperdiploid cells, as evaluated either *via* cytometry or fluorescence microscopy. Raw data regarding cell cycle analysis are available as **Supplementary Material**.

### CPZ Reduces GBM Cell Cloning Efficiency

A distinct hallmark of cancer cells, and especially GBM, is the ability to generate clones from cells seeded *in vitro* at elevated dilutions. We quantified the effect of CPZ in inhibiting cell cloning efficiency in GBM cells by using different methods, according to the capability of these cells to grow in an anchorage-dependent or -independent fashion.

**Figure 3:**
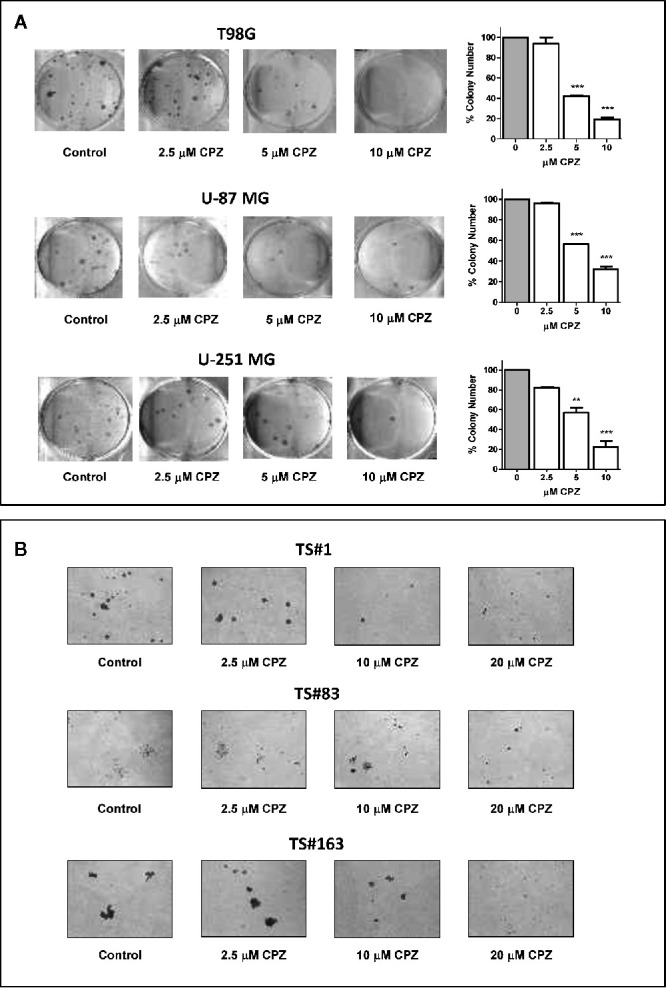
CPZ reduces cloning efficiency in GBM cells. **(A)**. Colony-forming assay. Anchorage-dependent cells were treated with increasing doses of CPZ, as indicated. At the end of the experiment, cells were stained and counted. The results are reported in the histogram on the far right of the panel along with statistical significance referred toward the Control (** p < 0.01; *** p < 0.001). **(B)**. Neurosphere formation assay. Cells were treated with increasing doses of CPZ, as indicated. Of note, TS#83 neurospheres displayed a partial anchorage-dependent growing capability. At the end of the experiment, neurospheres were photographed under an inverted microscope using a 4× objective.

CPZ drastically reduced colony number in T98G, U-251 MG, and U-87 MG anchorage-dependent cell lines. Colony number was also reported in a histogram for each experimental set (**Figure 3A**). In **Figure 3B**, a drastic drop in sphere dimensions and a clear impairment in sphere forming ability were also appreciable in the TS#1, TS#83, and TS#163 neurospheres, according to previous reports concerning other primary GBM cell lines (5, 32). The dose-dependent effect of CPZ in decreasing colony-forming efficiency in anchorage-dependent GBM cells, as well as in reducing the proficiency of neurospheres in forming 3D spheroids, strongly suggests the ability of this compound to inhibit clonogenic power, a common feature of cancer cells.

### CPZ Downregulates Stemness Gene Expression in GBM Cells

The sharp effect of CPZ in impairing neurosphere formation drove us to investigate the capability of this compound to reduce the stemness potential in stem-like GBM cells. Thus, we analyzed the relative expression of the stemness genes *OCT 3/4*, *SOX2*, *NANOG*, *Nestin*, *OLIG2* and *ALDH1A3* on TS#1, TS#83, and TS#163 neurospheres by means of qRT-PCR, using the primers listed in **Table 1**.

**Table 1:** Primers used for assaying the relative expression of the indicated stemness genes *via* qRT-PCR.

Primers	Sequence
OCT 3/4 FWD	5′-TGGAGAAGGAGAAGCTGGAGCAAAA-3′
OCT 3/4 REV	5′-GGCAGATGGTCGTTTGGCTGAATA-3′
SOX2 FWD	5′-CGATGCCGACAAGAAAACTT-3′
SOX2 REV	5′- CAAACTTCCTGCAAAGCTCC-3′
Nanog FWD	5′-CAAAGGCAAACAACCCACTT-3′
Nanog REV	5′-ATTGTTCCAGGTCTGGTTGC-3′
Nestin FWD	5′-CAGCGTTGGAACAGAGGTTGG-3′
Nestin REV	5′-TGGCACAGGTGTCTCAAGGGTAG-3′
OLIG2 FWD	5′-CCAGAGCCCGATGACCTTTTT-3′
OLIG2 REV	5′-CACTGCCTCCTAGCTTGTCC-3′
ALDH1A3 FWD	5′-TGAATGGCACGAATCCAAGAG-3′
ALDH1A3 REV	5′-CACGTCGGGCTTATCTCCT-3′
GAPDH FWD	5′-TCCCTGAGCTGAACGGGAAG- ′
GAPDH REV	5′-GGAGGAGTGGGTGTCGCTGT-3′

**Figure 4:**
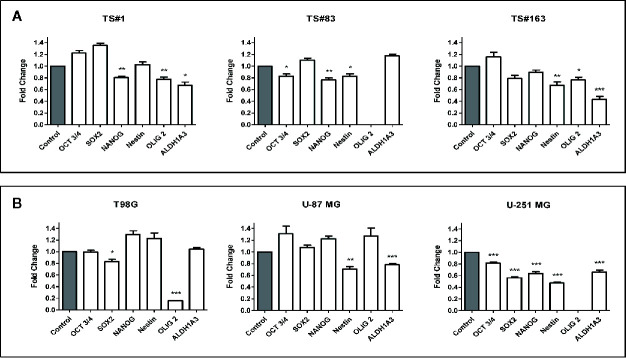
CPZ downregulates stemness gene expression in GBM cells. **(A)**. Expression of stemness genes in the three stem-like cell lines (neurospheres). **(B)**. Expression of stemness genes in the three anchorage-dependent cell lines. In all cases, determinations were performed *via* qRT-PCR after 24 h of exposure to CPZ. Histogram values represent the fold-changes referred to the respective value for untreated cells (Control), arbitrarily reported as 1.0 (gray columns on the left of each graph). Statistical significance is referred toward the Control (* p < 0.05; ** p < 0.01; *** p < 0.001). In TS#83 cells and U-251 MG cells, in panel A and in panel B respectively, the amount of OLIG2 mRNA was undetectable in Control as well in CPZ-treated cells.

Cells were exposed to CPZ for 24 h at the concentrations reported as IC30 in **Figure 1B**, or to an equal volume of solvent for the respective controls. As shown in **Figure 4A**, we observed a significant CPZ-induced reduction of the expression of selected stemness markers in neurospheres, showing a behavior peculiar for each cell line.

For the sake of completeness, we carried out the same assay also on the T98G, U-251 MG, and U-87 MG anchorage-dependent GBM cell lines, where the influence of CPZ on the expression of these stemness genes was clearly appreciable, especially in the U-251 MG cell line (**Figure 4B**).

It is worth noting that *ALDH* is a stem cell marker whose activity promotes tumorigenesis and progression in different solid tumors (33). In particular, the isoform *ALDH1A3* analyzed here promotes stemness, triggers mesenchymal transition in GBM and increases resistance to TMZ (34). CPZ significantly inhibited *ALDH1A3* gene expression in TS#1 TS#163, U-87 MG, and U-251 MG cell lines. *GAPDH* determination was employed for normalization purposes. Raw data regarding RT-PCR analysis are available as **Supplementary Material**.

### CPZ Synergizes With TMZ in Reducing GBM Cell Viability

**Figure 5:**
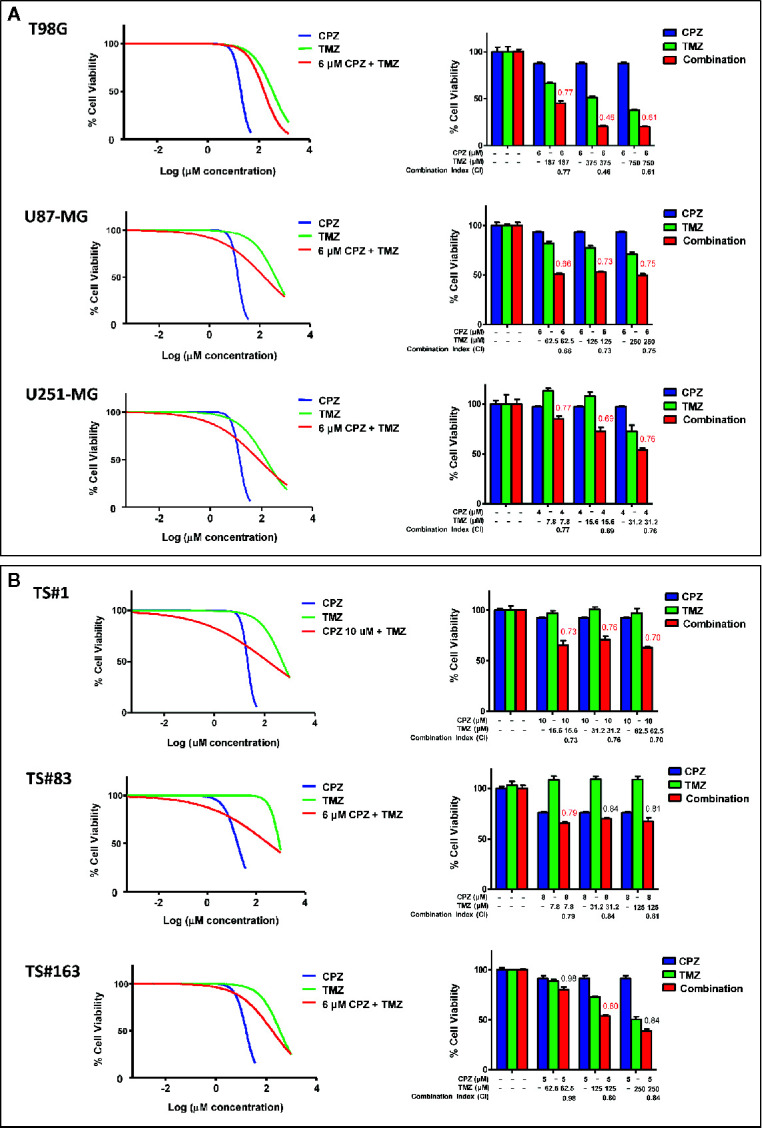
CPZ synergizes with TMZ in reducing GBM cell viability. Anchorage-dependent GBM cell lines **(A)** and neurospheres **(B)**. Dose–response effects of CPZ (blue), TMZ (green) and TMZ plus a constant (k) CPZ concentration, indicated for each cell line (red) on percent cell viability (left panels). Histograms show cell viability at selected drug concentrations, as indicated, to highlight the effect of the association of the two drugs (right panels). The effects of TMZ and CPZ combination were considered synergistic when the CI was <0.8 (in red). CPZ k values were 6.0, 6.0, 4.0, 10.0, 8.0 and 5.0 μM for T98G, U-87 MG, U-251 MG, TS#1, TS#83 and TS163 GBM cells, respectively.

Since CPZ restrained cell growth in all the GBM cell lines examined, we assayed its effect when administered in combination with TMZ, the first-line drug for GBM treatment. In **Figure 5**, the effects of CPZ alone (blue lines in graphs, blue columns in histograms) and TMZ alone (green lines in graphs, green columns in histograms) on cell viability are shown for the anchorage-dependent GBM cell lines and neurospheres (**Figure 5A, B**, respectively). In order to check for the effect of the combination of the two drugs, we exposed GBM cells to increasing doses of TMZ, ranging from 4 to 1,000 μM for 96 h prior the addition of CPZ, which was administered at a fixed concentration. CPZ doses were chosen in accordance with the individual sensitivity of each cell line to this compound and corresponded approximately to the IC10 (the amount of substance able to inhibit *in vitro* a given biological process by 10%) (see **Figure 5**). After 48 h of further incubation, cell viability was assessed. The effect of the drug combination is indicated for all the GBM cells assayed (red lines in graphs, red columns in histograms).

By using the algorithm described by Fransson et al. (28), we analyzed the outcome of the compounds used as single agents compared with the one of the drug combination, and expressed it as Combination Index (CI), whose value is reported on the top of the red columns. When the addition of CPZ to TMZ yielded a decrease in cell viability attributable to a synergistic effect of the two drugs (CI value <0.8), the respective CI value was reported in red. The combined effect of the two compounds was especially evident in the anchorage-dependent GBM cell lines and in TS#1 neurospheres, where drugs synergistically cooperated to reduce GBM cell viability. Raw data regarding cell viability analysis are available as **Supplementary Material**.

### CPZ Cooperates With TMZ in Inducing Cell Death

**Figure 6:**
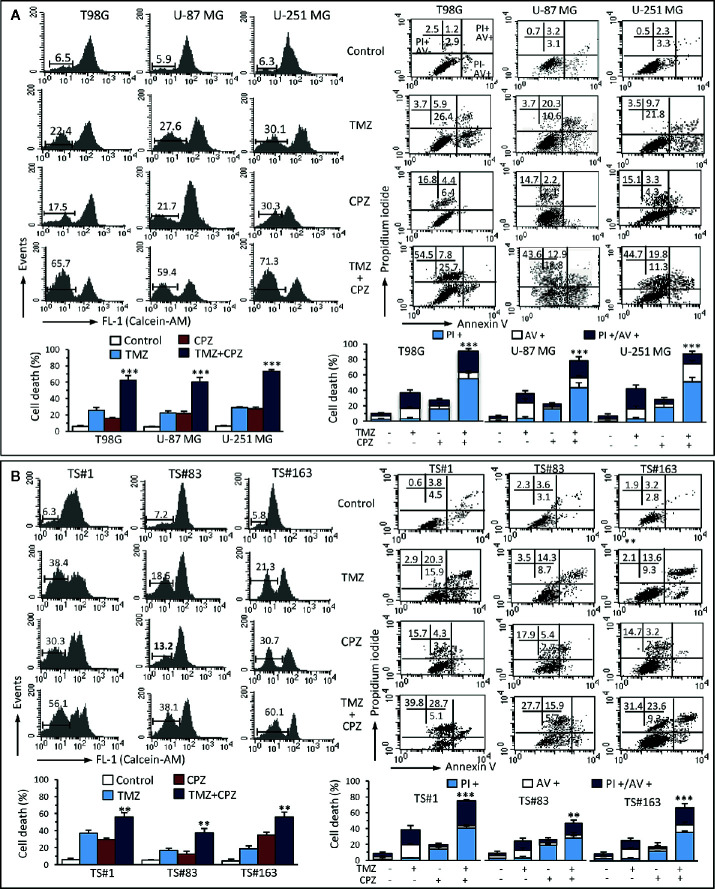
CPZ cooperates with TMZ in inducing cell death. Anchorage-dependent GBM cell lines **(A)** and neurospheres **(B)** were analyzed after treatment with TMZ (96 h) and CPZ (48 h), or their combination, at the lowest concentrations considered synergistic on the basis of the viability analysis. Left panels. FACS analysis after staining with Calcein-AM (which is retained in the cytoplasm of live cells). Numbers represent the percentage of Calcein-negative cells (dead cells). One representative experiment is shown. Bar graphs below show the results obtained from four independent experiments, reported as means ± SE. Right panels. FACS analysis after double staining with Annexin V/PI. Dot plots from a representative experiment are shown. Numbers represent the percentages of Annexin V-positive cells (bottom right quadrant), Annexin V/PI double positive cells (upper right quadrant), or PI-positive cells (upper left quadrant). Note the high percentage of cells positive for PI in CPZ treated cells. Bar graphs below show results obtained from four independent experiments, reported as means ± SE. ** p < 0.01 and *** p < 0.001 indicate significant differences *vs* single drug treatments (TMZ or CPZ).

With the aim of understanding the mechanisms elicited by TMZ, CPZ or their combination on GBM cells, we analyzed their effect on cell viability. Cytofluorimetric assay with Calcein-AM, coupled with Annexin V/PI analysis, allowed us quantifying cell death and apoptosis in the same experimental setup. The data reported in **Figure 6A**, for anchorage-dependent GBM cells indicate that cells treated with TMZ became positive to Annexin V, thus suggesting an apoptotic cell death, as previously described (35, 36). By contrast, CPZ appeared to induce toxicity *via* its ability to generate aberrant mitoses that cause nuclear fragmentation and cell death. Indeed, CPZ-treated cells were almost completely negative to Annexin V, but positive to PI.

We found similar results in neurospheres (**Figure 6B**) that, despite their positivity to Annexin V after treatment with TMZ, were resistant to classical caspase-mediated apoptosis, but died by ferroptosis, as we demonstrated earlier (37). Importantly, CPZ acted in cooperation with TMZ also in inducing death in neurospheres.

Analysis of cell death by Calcein-AM, a non-fluorescent dye converted to green-fluorescent calcein after acetoxymethyl ester hydrolysis by intracellular esterases in living cells, substantially confirmed data obtained by Annexin V/PI evaluation. Raw data regarding Annexin V and Calcein-AM analysis are available as **Supplementary Material**.

### CPZ Cooperates With TMZ in Reducing GBM Cell Cloning Efficiency

**Figure 7:**
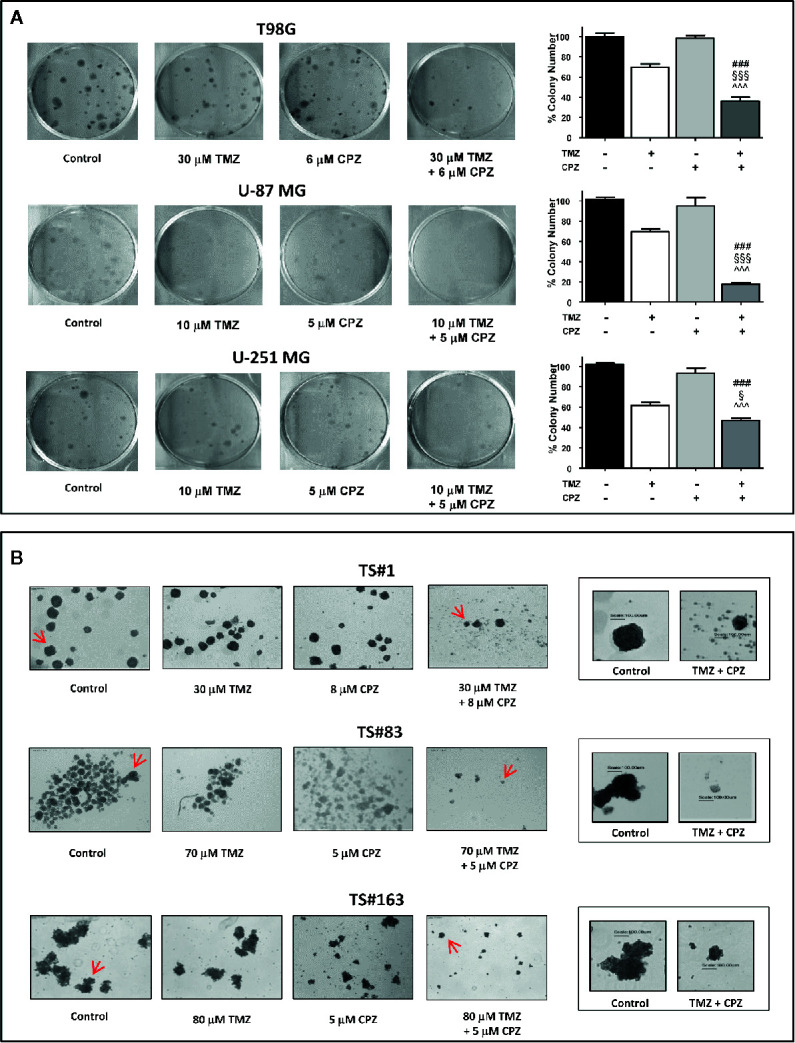
CPZ cooperates with TMZ in reducing GBM cell cloning efficiency. **(A)**. Anchorage-dependent cells T98G, U-87 MG and U-251 MG were exposed to solvent(s) (Control), TMZ, CPZ or their association at the doses indicated, then rinsed and allowed to grow in the absence of drugs for the subsequent 12 d. Cell colonies, after staining with crystal violet (left), were counted, and the values reported as percent colony number in the histogram (right). In these panels, variance among groups was assessed *via* the Bartlett’s test for equal variances. Statistical analysis among groups was done using the One-way ANOVA test followed by the Tukey’s Multiple Comparison Test (### significance <0.001 vs. Control; ^^^ significance <0.001 vs. CPZ; §§§ significance <0.001 vs. TMZ; § significance <0.05 vs TMZ). **(B)**. Neurospheres TS#1, TS#83, and TS#163 were treated as described and allowed to grow and form spheres for the subsequent 20 d. For each cell line, the four left images illustrate the effect of TMZ, CPZ or both drugs on neurosphere number and size, observed using a 4× objective. The two framed images on the right are enlarged pictures of the respective neurospheres indicated by the red arrows; these neurospheres were randomly chosen due to their dimensions, in order to appreciate their decrease in volume due to the treatment with the drug combo. This suggests a reduced sphere-forming ability in TMZ + CPZ-treated cells. In this panel, no histograms with values and statistical significance are reported, due to the intrinsic difficulty of objectively counting floating neurospheres.

Anchorage-dependent GBM cell lines exposed to a combination of TMZ and CPZ drastically reduced their cloning efficiency. Colony number, quantification of the experimental results and their statistical significance are represented in the histogram on the right of each experimental set (**Figure 7A**).

Then, we analyzed the sphere-forming efficiency in neurospheres treated with sub-optimal doses of TMZ and/or CPZ. While administration of these drugs as a single compound did not reach a noticeable effect, the same doses, administered in combination, produced a marked decrease in both sphere number and volume (**Figure 7B**). This last feature was particularly evident in the right panels (enlarged), where a scale bar allowed the comparison of the size between representative control and TMZ plus CPZ-treated neurospheres.

Colony- and sphere-forming ability assays demonstrate that CPZ cooperated with TMZ in reducing GBM cell cloning efficiency, a distinctive signature of malignancy in cancer cells.

## Discussion

The current therapeutic protocol for newly diagnosed GBM results in suboptimal clinical outcomes. In this urgent need for novel therapeutic strategies, scientifically supported drug repurposing represents an appealing alternative, since it involves the use of compounds with shorter development timelines and lower risks for the patients, allowing faster and less expensive delivery from bench to bedside of potentially effective drugs. In the oncology field, several non-cancer drugs have been proposed and employed in clinical trials for GBM patients (38). Among these, CPZ has been shown effective in hindering key biological features of cancer cells *in vitro*, also in the case of malignant gliomas (15).

Here we confirm the ability of CPZ in restraining key cancer cell features, adding further information concerning the effect of the drug on six human GBM cell lines, either anchorage-dependent or patient-derived neurospheres. In all these cell lines, CPZ generated nuclear aberrations that, associated with its ability to protect from apoptosis, drove cells toward anomalous mitoses, possibly *via* the described inhibition of the mitotic kinesin KSP/Eg5 (8), with subsequent death to be expected *via* mitotic catastrophe. In the same experimental setting, the RPE-1 non-cancer cell line resulted less sensitive to the drug, when compared with the anchorage-dependent GBM cells and, remarkably, refractory to the induction of nuclear aberrations elicited by the drug, as detected in all the GBM cells assayed. These last results need to be validated using other non-cancer model systems; indeed, while the reasons for this selectivity have not been identified yet, such evidence could be of considerable interest in GBM therapy.

In addition, CPZ was able to downregulate the expression of *OCT 3/4*, *SOX2*, *NANOG*, *Nestin*, *OLIG2* and *ALDH1A3*, universal cancer stem cells genes for GBM (39). In particular, *ALDH1A3* expression is related to resistance to TMZ (34). Specifically, stemness characteristics in GBM appear related to the expression of updated gene sets, especially when validated in detailed culture conditions (organoids) and/or *in vivo* (40). It is also worth mentioning that CPZ strongly affected GBM cloning efficiency and neurosphere-forming capability, two features of remarkable therapeutic relevance, since stem cells are considered responsible for GBM drug resistance and clinical relapse (41, 42).

Noteworthy, the first described and most investigated feature of CPZ is the inhibition of the dopamine receptor DRD2 [see (43) for a review]. More recently, this compound has also been identified, together with some homologs, as an inhibitor of AMPA and NMDA glutamate receptor channels (44). Dopamine, glutamate and their receptors are vital for physiological neuronal synaptic signaling, and the recent identification of neuron-glioma synapses is giving high relevance to the role of these neuromediators and their post-synaptic receptors in brain cancer proliferation and progression (45, 46). Therefore, the pharmacological effects of CPZ on these receptors might play a major role in brain cancer therapy.

The state of the art of GBM chemotherapy relies on TMZ, a drug proficient in inhibiting GBM growth *in vitro*, even if the doses required to reach the IC50 in these cells result quite high (19) and not comparable at all with those reachable *in vivo*. TMZ, as an alkylating agent, damages guanine residues of DNA. These damages are partly recovered in cells expressing adequate levels of the O-6-methylguanine-DNA methyltransferase (*MGMT*) gene. Since expression, and thus activity, of the related protein is prevented in some GBM cells by methylation of the promoter of this gene, GBM cells displaying hypo- or non-methylated *MGMT* gene express more MGMT protein, thus being less sensitive to TMZ, causing an intrinsic or acquired resistance to this drug.

In order to provide a rationale for a clinical trial for GBM patients, we evaluated the effect of the combination of CPZ plus TMZ on selected cellular parameters, demonstrating a clear synergism between these two drugs in reducing cell viability and a sharp cooperation in restraining cloning efficiency and neurosphere formation. Moreover, by combining two different quantitative assays, Calcein-AM and Annexin V/PI, we highlighted how these drugs induced cell death *via* distinct mechanisms. The different mode of action exerted by these drugs might explain their ability to cooperate in inducing cell death and thus restraining GBM growth in both anchorage-dependent cells and neurospheres. Indeed, while TMZ is known to block the DNA replication fork, thus arresting the cell cycle in the G2/M phase, [see **Figure 2** and refs. (35, 36)], CPZ, besides its ability to hinder GBM cells at the G2/M boundary (see **Figure 2**), appears capable of protecting aberrant or defective cells from apoptosis. Such a feature would drive these cells towards a mitosis with very little chances of being completed, eliciting, on the contrary, the generation of daughter cells with abnormal chromosomal makeup [see **Figure 2** and ref (8)] and death by mitotic catastrophe. It has been demonstrated that autophagic cell death contributes to CPZ-induced cytotoxicity in GBM cells (10). On these bases, the combination of TMZ plus CPZ would simultaneously trigger apoptosis, ferroptosis (especially in neurospheres), mitotic catastrophe, and autophagic death in GBM cells, synergistically increasing the cytotoxic effect in GBM and contributing to overcome drug resistance.

GBM is a disease characterized by profound intra-tumor heterogeneity (47) and hierarchically dependent upon pre-existing cancer stem cells that undergo selection under therapeutic pressure (48), with a consequent unpredictable genetic drift (2). Such a scenario implies an intrinsic challenge in the choice of a suitable targeted therapy. From the scientific literature and our results, CPZ appears as a drug with multifaceted effects on cancer cells, being able to affect major signal transduction pathways, spindle assembly and apoptosis, all processes conceivably essential for the survival of the different clones that characterize the marked GBM heterogeneity.

CPZ is the progenitor of the DRD2 inhibitors phenothiazines and is used since the 50s in the therapy of several psychiatric disorders, *e.g.* acute and chronic psychosis, and provides relief from severe vomiting and untreatable hiccups. When necessary, CPZ can be administered for long periods, with doses ranging from 50 to 400 mg/day. Its main side effects are a dose-dependent sedation and, at higher doses, the occurrence of an extrapyramidal syndrome, both reversible when the drug is suspended. Presently, second and third generation neuroleptic drugs acting as DRD2 inhibitors are available, but we focused on CPZ since it is included in the 2019 World Health Organization Model List of Essential Medicines (49). The adverse reactions elicited by CPZ should not impede the treatment of GBM patients, also considering the poor prognosis attainable, especially in subjects carrying a GBM with a hypo- or non-methylated *MGMT* gene. In addition, CPZ crosses easily the blood-brain barrier.

In the light of these considerations, we submitted a Phase II clinical trial to our Institutional Ethical Committee (Comitato Etico Centrale IRCCS-Sezione IFO-Fondazione Bietti, Rome, Italy), which was approved on September 6, 2019 (EudraCT # 2019-001988-75; ClinicalTrials.gov Identifier: NCT04224441). The schedule consists in the addition of CPZ to the standard GBM treatment in patients carrying hypo- or un-methylated *MGMT* gene, *i.e.* those more resistant to TMZ. CPZ is administered orally at the dose of 50 mg/day, in concomitance with the adjuvant treatment with TMZ.

We are currently investigating, *via* high-throughput methodologies, the effects of CPZ on GBM cell signaling and energy metabolism, as well as identifying, *via* mass spectrometry, other relevant cellular factors directly targeted by the drug.

We expect that our *in vitro* results on the effectiveness of CPZ in restraining GBM cell growth, as well as its synergism with TMZ, could be replicated in the ongoing clinical setting. In addition to such a desirable outcome, which remains the main goal of our efforts, the use of a repurposed medication would allow reducing both development expenses and time predictable for a new drug to travel from the experimental laboratory to the clinics.

## Data Availability Statement

The datasets presented in this study can be found in online repositories. The names of the repository/repositories and accession number(s) can be found in the article/**Supplementary Material**.

## Author Contributions

CA, MGP, PM, AP and SM designed the study;, CA, SM, PM, BA, LRV and MB applied specific methodologies; CA, SM, PM and BA validated, the data; formal analysis, CA, SM, PM and BA; investigation, CA, SM, PM and BA; resources, CA, SM, PM, BA LRV, MB, RP, VV and AP; data curation, CA, SM, PM and BA; writing—original draft preparation, MGP, CA, SM and PM; writing—review and editing, MGP, CA, SM, PM, AP and VV; supervision, CA and MGP; funding acquisition, PM, RP and MGP. All authors contributed to the article and approved the submitted version.

## Funding

This work has been partially funded by Associazione Italiana per la Ricerca sul Cancro (IG 18526 to PM and 23154 to RP), Arcobaleno Onlus and Peretti Foundation (NaEPF 2019-042) to PM; Ricerca Corrente IRE 2018-2019 to MP.

## Conflict of Interest

The authors declare that the research was conducted in the absence of any commercial or financial relationships that could be construed as a potential conflict of interest.

## Supplementary Material

All Supplementary Material can be found as raw data at the link below: https://gbox.garr.it/garrbox/index.php/s/ip0LBxGQDns2qMs.

## Abbreviations

GBM, glioblastoma; CPZ, chlorpromazine; TMZ, temozolomide; DRD2, brain dopamine receptor D2; RPE-1, retinal pigment epithelial cells; AMPA receptor, α-amino-3-hydroxy-5-methyl-4-isoxazolepropionic acid receptor; NMDA receptor, *N*-methyl-D-aspartate receptor; MGMT, O-6-methylguanine-DNA methyltransferase.
